# *C*-mannosyl tryptophan dynamics in a mouse model of the peritoneal dissemination of ovarian cancer

**DOI:** 10.1016/j.jbc.2026.111359

**Published:** 2026-03-09

**Authors:** Yoko Inai, Shiho Minakata, Kaya Tsujimoto, Shino Manabe, Naoyuki Iwahashi, Ryota Kamijo, Yuma Nakadaira, Keisuke Nishikawa, Tomohiro Hashizume, Kazuhiko Ino, Yoshito Ihara

**Affiliations:** 1 Department of Biochemistry, School of Medicine, Wakayama Medical University, Wakayama, Japan; 2 School of Pharmacy and Pharmaceutical Sciences, Hoshi University, Tokyo, Japan; 3 Research Center for Pharmaceutical Development, Graduate School of Pharmaceutical Science & Faculty of Pharmaceutical Sciences, Tohoku University, Miyagi, Japan; 4 Department of Obstetrics and Gynecology, School of Medicine, Wakayama Medical University, Wakayama, Japan

**Keywords:** *C*-mannosyl tryptophan, *C*-mannosylation, glycosylation, macrophage, ovarian cancer, post-translational modification (PTM), protein degradation, thrombospondin

## Abstract

*C*-Mannosyl tryptophan (*C*-Man-Trp), a unique monomeric glycosyl amino acid, is up-regulated in the blood of ovarian cancer patients; however, the underlying mechanisms remain unclear. In the present study, *C*-Man-Trp production and its dynamics were investigated in female B6C3F1 mice transplanted with mouse ovarian cancer OV2944-HM-1 (HM-1) cells. After transplantation, *C*-Man-Trp levels increased in the plasma, urine, ascites, peritoneal exudate cells (PECs), and tumor masses of mice. Furthermore, changes in the transcriptional expression of *C*-Man-Trp metabolism-related genes, *C*-mannosyltransferases (*Dpy19l1* and *Dpy19l3*), and thrombospondin type I repeat superfamily genes (*Thbs1*, *Spon1*, and *cellular communication network factor 1*) were noted in tumor-associated cells and tissues. A cell-sorting analysis revealed that PECs mainly comprised myeloid-derived immune cells, such as macrophages and myeloid-derived suppressor cells, in addition to a small population of HM-1 tumor cells. *C*-Man-Trp levels were high in the macrophage fraction, but lower in the myeloid-derived suppressor cell fraction. *C*-Man-Trp was also produced in the *ex vivo* culture medium of macrophages isolated from PECs. Under macrophage depletion using clodronate liposomes, the ovarian cancer-stimulated up-regulation of *C*-Man-Trp was significantly suppressed in the plasma, ascites, PECs, and tumor masses of HM-1 cell-transplanted mice. *C*-Man-Trp levels in the plasma and peritoneal cavity cells of normal healthy mice were also suppressed by clodronate liposomes, whereas the expression of *C*-Man-Trp metabolism-related genes showed different changes from those in mice transplanted with HM-1 cells. Collectively, these results demonstrate that tumor-stimulated macrophages play a pivotal role in the dynamics of *C*-Man-Trp in mice with ovarian cancer.

*C*-Mannosyl tryptophan (*C*-Man-Trp) is a glycosyl amino acid in which tryptophan is conjugated with a single mannose *via* a C-C bond (1, 2, 3). A monomer form of *C*-Man-Trp was initially detected in human urine (4), and the structure of *C*-Man-Trp was also identified as a post-translational modification in the secretory protein ribonuclease 2 (1). The *C*-mannose attachment to tryptophan reportedly occurs at the N-terminal tryptophan in the consensus amino acid motif Trp-X-X-Trp/Cys in proteins (3, 5, 6). The *C*-mannose attachment is considered to be produced by a *C*-mannosyltransferase, with which an α-mannose is transferred from dolichylphosphate mannose to the indole C2 atom of the tryptophan of proteins in the endoplasmic reticulum of cells (7). Mammal *C*-mannosyltransferase genes were identified as *DPY19L1* (6) and *DPY19L3* (5), which exhibit distinct enzyme specificities. Although the metabolism of the monomeric form of *C*-Man-Trp has yet to be examined in detail, several lines of evidence indicate that at least some *C*-Man-Trp is derived from the degradation of *C*-mannosylated proteins in cells (8). In addition to its production, *C*-Man-Trp may be degraded by some bacteria species and used as a carbon source (9). However, the catabolic process for monomeric *C*-Man-Trp in higher organisms remains unknown.

*C*-Man-Trp is present in serum or plasma and is excreted in urine, and markedly higher concentrations of *C*-Man-Trp have been reported in the urine than in the blood of mice (10). Elevated *C*-Man-Trp levels in blood have been detected in patients with kidney dysfunction (11, 12, 13, 14, 15, 16, 17, 18, 19, 20), potentially reflecting a decline in the filtration function of the kidneys. A metabolomic analysis of serum was recently conducted in a broad range of biochemical investigations, and the findings obtained implicated changes in *C*-Man-Trp concentrations in serum or plasma with a number of pathological conditions other than kidney dysfunction, such as cardiovascular disease mortality (21), vascular complications (18), myeloproliferative neoplasms with thrombocytosis (22), and aging (23). These findings suggest the involvement of *C*-Man-Trp metabolism in a number of pathophysiological processes in humans. In other words, *C*-Man-Trp may serve as an indicator of the dynamics of protein *C*-mannosylation metabolism in human health and disease. However, the mechanisms by which *C*-Man-Trp metabolism changes under each pathophysiological condition have yet to be investigated. Furthermore, where and how *C*-Man-Trp is produced in body fluids, such as blood, in the bodies of higher organisms remain unknown.

We previously examined the tissue distribution of *C*-Man-Trp in mice, and found that female reproductive organs, such as the ovary and uterus, contained high levels of *C*-Man-Trp (10). This prompted us to measure plasma *C*-Man-Trp levels in ovarian cancer patients, and our findings revealed that plasma *C*-Man-Trp levels correlated with the malignancy of ovarian cancer, and also implicated *C*-mannosylated protein metabolism in the development of ovarian cancer (24). However, it remains unclear whether and how *C*-Man-Trp metabolism and dynamics change in ovarian cancer patients.

The *C*-mannosylated proteins identified to date are mostly secretory or membrane proteins, which are mainly members of the thrombospondin type I repeat (TSR) superfamily or cytokine receptor type-1 family (3, 25). Several TSR superfamily proteins with *C*-mannosylation, such as thrombospondin 1 (TSP1) and spondin 1/F-spondin, are cancer-related proteins (26, 27, 28, 29), and have been shown to affect cell proliferation, migration, attachment, or angiogenesis. Therefore, *C*-mannosylated TSR superfamily proteins may be involved in the regulation of pathophysiological processes in ovarian cancer. Based on these findings, further studies are warranted on the dynamic relationship between *C*-Man-Trp and *C*-mannosylated substrate proteins in ovarian cancer.

We herein investigated the production and dynamics of *C*-Man-Trp inside the ovarian tumor-bearing bodies of a mouse model of the peritoneal dissemination of ovarian cancer OV2944-HM-1 (HM-1) cells. We identified the source of *C*-Man-Trp production in tumor-induced peritoneal cells and elucidated the *in vivo* dynamics of *C*-Man-Trp with changes in the expression of *C*-Man-Trp metabolism-related molecules, such as *C*-mannosyltransferases and TSR superfamily proteins, in mice with ovarian cancer.

## Results

### *C*-Man-Trp production increased in the bodies of mice with ovarian cancer

Mouse ovarian cancer HM-1 cells were transplanted into the peritoneal cavities of female B6C3F1 mice, as described in Experimental procedures. The peritoneal metastasis model of HM-1 cells has been widely accepted for investigating the peritoneal dissemination of ovarian cancer (30, 31). We examined the effects of the peritoneal dissemination of ovarian cancer on *C*-Man-Trp production and dynamics in mice.

**Figure 1:**
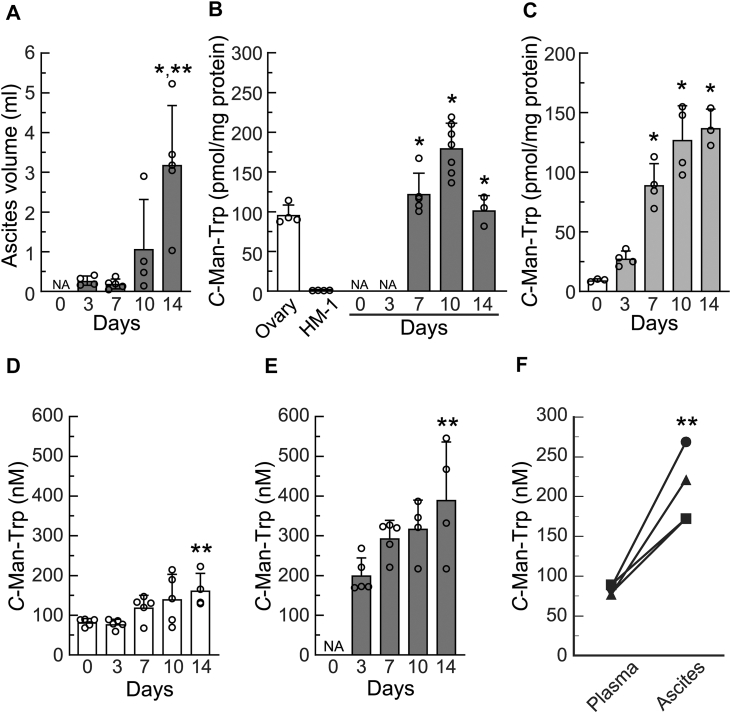
***C*-Man-Trp levels increased in mice with ovarian cancer.** OV2944-HM-1 (HM-1) cells were transplanted into the peritoneal cavities of female B6C3F1 mice as described in the Experimental procedures. After the transplantation of HM-1 cells, mice were sacrificed at the indicated time points, and blood, ascites, tumor masses, and peritoneal tissues were collected for examination. Normal ovary tissues and HM-1 cells under *in vitro* culture conditions were also tested. *A*, the ascites volume in mice was measured at the indicated time points. NA; not applicable. ∗*p* < 0.01 *versus* days 3 and 7, ∗∗*p* < 0.05 *versus* day 10. *B*, *C*-Man-Trp levels in normal mouse ovarian tissues, HM-1 cells, and tumor mass tissues were examined. NA; not applicable. ∗*p* < 0.01 *versus* HM-1. *C*, *C*-Man-Trp levels in peritoneal tissue samples were examined. ∗*p* < 0.01 *versus* day 0. *D*, *C*-Man-Trp levels in plasma samples were examined. ∗∗*p* < 0.05 *versus* days 0 and 3. *E*, *C*-Man-Trp levels in ascites samples were examined. NA; not applicable. ∗∗*p* < 0.05 *versus* day 3. F, *C*-Man-Trp levels were plotted and compared for plasma and ascites samples from identical mice on day 3 after HM-1 cell transplantation. ∗∗*p* < 0.05 *versus* plasma.

Ascites was produced and detected in the peritoneal cavity 3 days after the transplantation of ovarian cancer cells and its volume gradually increased over 14 days (Fig. 1*A*). Tumor masses were detected in the peritoneal cavity 7 days after transplantation and contained high levels of *C*-Man-Trp (Fig. 1*B*). *C*-Man-Trp levels in the tumor masses were similar or slightly higher than those in normal ovarian tissues, but were significantly higher than those in HM-1 cells, which were used for tumor seeding. Peritoneal tissues in healthy mouse contained low *C*-Man-Trp levels, which gradually increased over 10 days after tumor transplantation (Fig. 1*C*). In the plasma of tumor-bearing mice, *C*-Man-Trp levels gradually increased in a time-dependent manner and significantly increased 14 days after transplantation (Fig. 1*D*). A high level of *C*-Man-Trp was detected in ascites 3 days after transplantation and was maintained over 14 days (Fig. 1*E*). Comparisons of *C*-Man-Trp levels in plasma and ascites from identical individuals on day 3 after transplantation revealed significantly higher levels in the latter than in the former (Fig. 1*F*).

**Figure 2:**
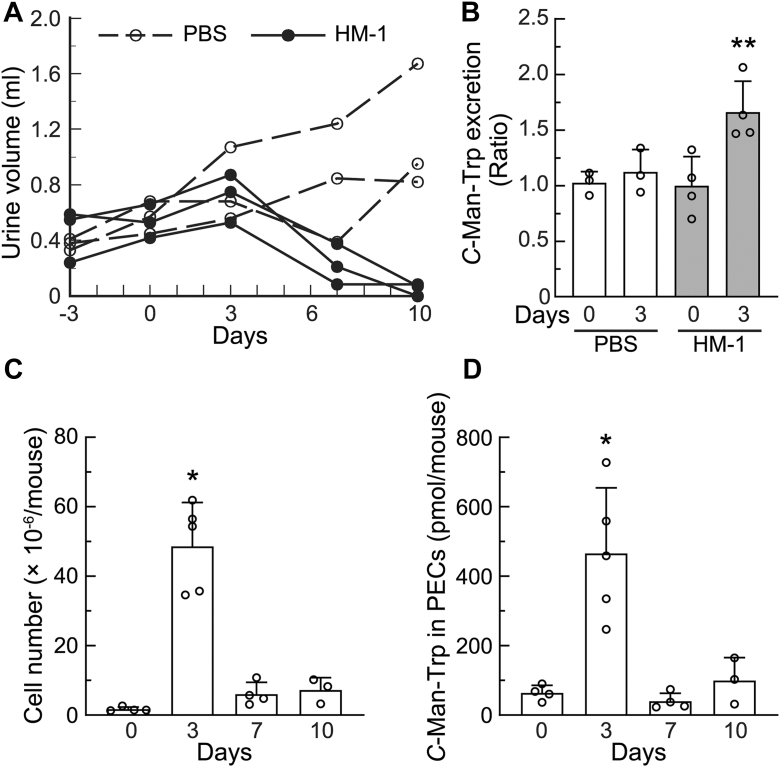
***C*-Man-Trp levels increased in the urine and peritoneal exudate cells of mice with ovarian cancer.** HM-1 cells were transplanted into the peritoneal cavities of female mice as in Figure 1. Female mice with PBS injected into the peritoneal cavity were used as controls. *A*, urine volume was measured at the indicated time points after the transplantation of HM-1 cells. *B*, the urinary excretion of *C*-Man-Trp was examined in mice on days 0 and 3 after the injection with PBS or HM-1 cells. ∗∗*p* < 0.05 *versus* HM-1 (day 0). *C* and *D*, after the injection of HM-1 cells, mice were sacrificed at the indicated time points, and peritoneal exudate cells (PECs) were collected. The number of PECs in mice was shown (*C*). The PECs were also subjected to *C*-Man-Trp measurements as described in the Experimental procedures (*D*). ∗*p* < 0.01 *versus* days 0, 7, and 10.

Urine volume was also examined in mice with and without HM-1 cell transplantation. As shown in Figure 2*A*, urine volume was not markedly affected until day 3 after transplantation, and no significant changes were observed in plasma creatinine levels (day 0, 0.108 ± 0.015 mg/dl; day 3, 0.096 ± 0.009 mg/dl), indicating normal renal function in the early phase. However, 7 days after transplantation, urine volume was markedly lower than that in the controls, suggesting a urinary excretion disorder due to tumor transplantation. Therefore, we examined *C*-Man-Trp levels excreted in urine 3 days after the transplantation of HM-1 cells. As shown in Figure 2*B*, *C*-Man-Trp levels excreted in urine were significantly higher on day 3 after the transplantation of HM-1 cells than in the control (*i.e.*, PBS).

We focused on a cellular fraction that was distinct from tumor masses in the peritoneal cavities of mice after HM-1 cell transplantation. Intraperitoneal floating cells were collected by centrifugation using conventional conditions for cultured cells (*i.e.*, 800*g* for 5 min), and were named peritoneal exudate cells (PECs). In Figure 2*C*, the number of PECs in healthy mice was at least 1 × 10^6^ on day 0 and then markedly increased, reaching a maximum on day 3 after transplantation. In Figure 2*D*, a high *C*-Man-Trp level was also detected in PECs on day 3. Therefore, *C*-Man-Trp levels appeared to increase in various cellular fractions, such as tumor masses, peritoneal tissues, and PECs, in mice transplanted with HM-1 cells. Furthermore, extracellular levels of *C*-Man-Trp increased in various body fluids, such as plasma, ascites, and urine, in mice with ovarian tumors. Collectively, these results suggest that *C*-Man-Trp levels increased throughout the whole bodies of mice with ovarian cancer.

### The transcriptional expression of C-mannosyltransferases and TSR superfamily proteins increased in peritoneal tissues and PECs in mice with ovarian cancer

HM-1 cells were transplanted into the peritoneal cavities of female mice, and tumor-associated tissue samples were collected 7 days later. *C*-Man-Trp metabolism and dynamics are considered to be controlled by the production and degradation of *C*-mannosylated proteins in cells (25). Therefore, we focused on *C*-Man-Trp metabolism-related molecules, such as *C*-mannosyltransferases and TSR superfamily proteins, and examined the transcriptional expression of the selected molecules in the peritoneal tissues, PECs, and tumor mass tissues of mice with ovarian tumors using reverse-transcription quantitative PCR (RT-qPCR). mRNA levels were indicated as relative values to those of normal ovarian tissues.

**Figure 3:**
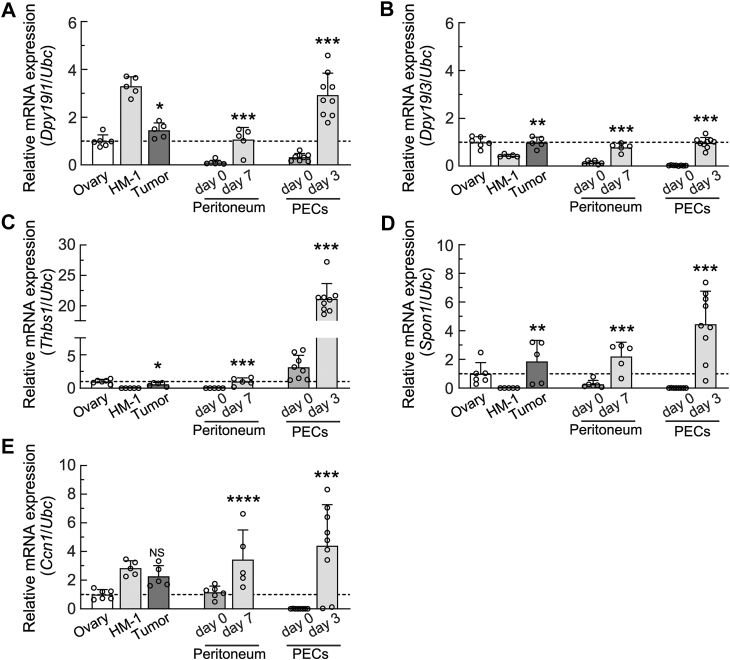
**The transcriptional expression of *C*-mannosyltransferases and thrombospondin type I repeat superfamily proteins in mice with ovarian cancer.** HM-1 cells were transplanted into the peritoneal cavities of female mice as in Figure 1. Total RNA was extracted from tumor masses (day 7), PECs (days 0 and 3), and peritoneal tissues (day 0), and samples were then subjected to RT-qPCR as described in the Experimental procedures. Total RNA was also extracted from normal mouse ovarian tissues and HM-1 cells under *in vitro* culture conditions, and samples were then subjected to RT-qPCR analyses. *Ubc* mRNA was used as the reference for the normalization. mRNA levels were indicated as relative values to those of normal ovarian tissues. A, *Dpy19l1*. B, *Dpy19l3*. *C*, *Thbs1*. *D*, *Spon1*. *E*, *cellular communication network factor 1*. ∗*p* < 0.01, ∗∗*p* < 0.05, NS; not significant *versus* HM-1. ∗∗∗*p* < 0.01, ∗∗∗∗*p* < 0.05 *versus* day 0. RT-qPCR, reverse-transcription quantitative PCR; *Ubc*, ubiquitin.

Regarding *C*-mannosyltransferases (DPY19L1 and DPY19L3), the transcriptional level of *Dpy19l1* was higher in HM-1 cells than in normal ovarian tissues and tumor mass tissues (Fig. 3*A*). *Dpy19l1* levels were low in peritoneal tissues and PECs under untreated conditions (day 0), but significantly increased in peritoneal tissues with metastatic lesions on day 7 after transplantation or in PECs on day 3 after transplantation. *Dpy19l1* levels were >9-fold higher in PECs (day 3) than in the untreated control. In contrast, the transcriptional level of *Dpy19l3* was lower in HM-1 cells than in normal ovarian tissues and tumor mass tissues (Fig. 3*B*). In peritoneal tissues and PECs, *Dpy19l3* levels increased after transplantation to those observed in normal ovarian tissues. These results indicate that the expression of both *C-*mannosyltransferases was significantly up-regulated in peritoneal tissues and PECs from mice after transplantation.

Regarding TSR superfamily proteins, we selected several ovarian cancer-related proteins, such as TSP1 (32, 33), spondin 1 (34, 35), and cellular communication network factor 1 (CCN1)/cysteine-rich angiogenic inducer 61 (36, 37), for further analyses. In the case of TSP1, the transcriptional level of *Thbs1* was negligible in HM-1 cells, but was similar between normal ovarian tissues and tumor mass tissues (Fig. 3*C*). In peritoneal tissues, the level of *Thbs1* was also lower under untreated conditions (day 0) than in normal ovarian tissues. The level of *Thbs1* significantly increased on day 7 after transplantation to that in normal ovarian tissues. In PECs, the level of *Thbs1* (day 0) was higher than that in normal ovarian tissues, and significantly increased on day 3 after transplantation.

Regarding spondin 1, the transcriptional level of *Spon1* was negligible in HM-1 cells and similar between normal ovarian tissues and tumor mass tissues (Fig. 3*D*). In peritoneal tissues, *Spon1* levels were low on day 0 and significantly increased on day 7 after transplantation. In PECs, *Spon1* levels were also negligible on day 0, but were significantly higher on day 3 after transplantation than in untreated controls (day 0). In the case of CCN1, the transcriptional levels of *Ccn1* in HM-1 cells and tumor mass tissues were higher than in normal ovarian tissues (Fig. 3*E*). In peritoneal tissues, the level of *Ccn1* under untreated conditions (day 0) was similar to that in normal ovarian tissues, and significantly increased on day 7 after transplantation. In PECs, the level of *Ccn1* was negligible on day 0, but significantly increased on day 3 after transplantation.

These results demonstrated that the transcriptional expression of *Dpy19l1*, *Dpy19l3*, *Thbs1*, *Spon1*, and *Ccn1* was up-regulated in peritoneal tissues with metastatic lesions and PECs in mice with ovarian cancer. Regarding *Thbs1* in PECs, its transcriptional expression was significantly up-regulated by the transplantation of HM-1 cells. These results revealed changes in *C*-Man-Trp metabolism and dynamics in the peritoneal cavities of mice with ovarian cancer.

### TSP1 protein levels decreased in PECs from mice with ovarian cancer

**Figure 4:**
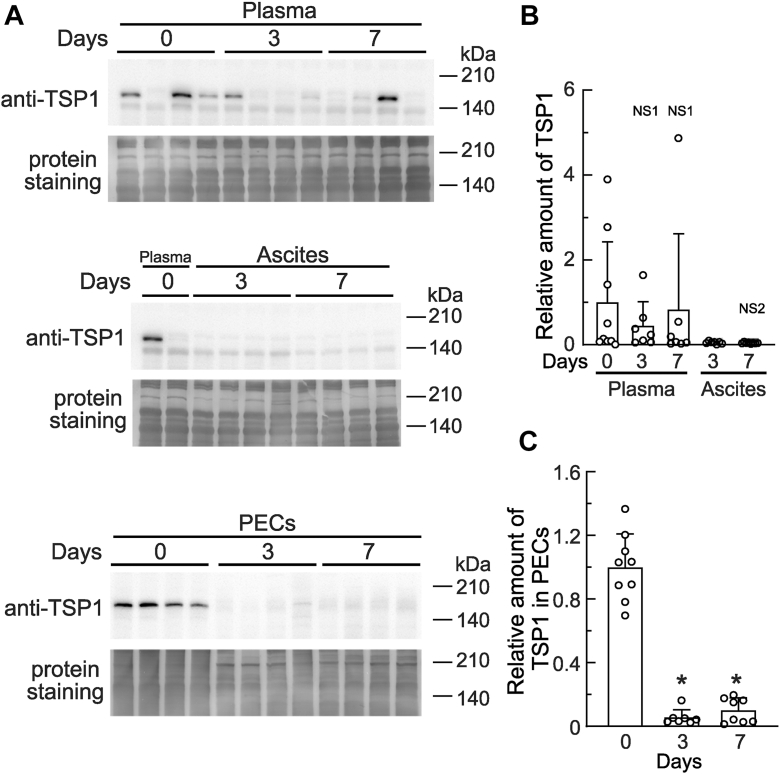
**TSP1 levels in plasma, ascites, and peritoneal exudate cells from mice with ovarian cancer.** HM-1 cells were transplanted into the peritoneal cavities of female mice as in Figure 1, and samples of plasma, ascites, and PECs were collected at the indicated times post-transplantation. *A*, TSP1 levels in plasma, ascites, and PECs were examined using an immunoblot analysis with the anti-TSP1 antibody. Total proteins on the membranes were stained as described in the Experimental procedures, and used for the loading controls. *B*, TSP1 levels in plasma and ascites were quantified and shown. NS1, not significant *versus* plasma (day 0). NS2, not significant *versus* ascites (day 3). *C*, TSP1 levels in PECs were quantified and shown. ∗*p* < 0.01 *versus* day 0. TSP1, thrombospondin 1.

Based on the results shown in Figure 3*C*, we investigated TSP1 protein expression in the bodies of mice with ovarian cancer. After the transplantation of HM-1 cells, biological samples (*i.e.*, plasma, ascites, and PECs) were taken on the days indicated, and TSP1 levels were assessed by immunoblotting. In Figure 4, *A* upper and *B*, TSP1 protein levels in plasma varied considerably among mice. However, the levels were similar on day 0 to those after transplantation. TSP1 was also detected in ascites, but its level was lower than that in plasma. TSP1 levels in ascites were similar between days 3 and 7 after transplantation (Fig. 4, *A* middle and *B*). On the other hand, TSP1 was detected in PECs on day 0, and its level significantly decreased on days 3 and 7 after transplantation (Fig. 4, *A* bottom and *C*), despite the transcriptional expression of *Thbs1* being significantly up-regulated on day 3 after transplantation (Fig. 3*C*). These results suggest that, in the peritoneal cavities of mice with ovarian cancer, the degradation of TSP1 was enhanced in PECs and/or its extracellular space.

### PECs from mice with ovarian cancer mainly comprised myeloid lineage cells containing macrophages and myeloid-derived suppressor cells (MDSCs)

**Figure 5:**
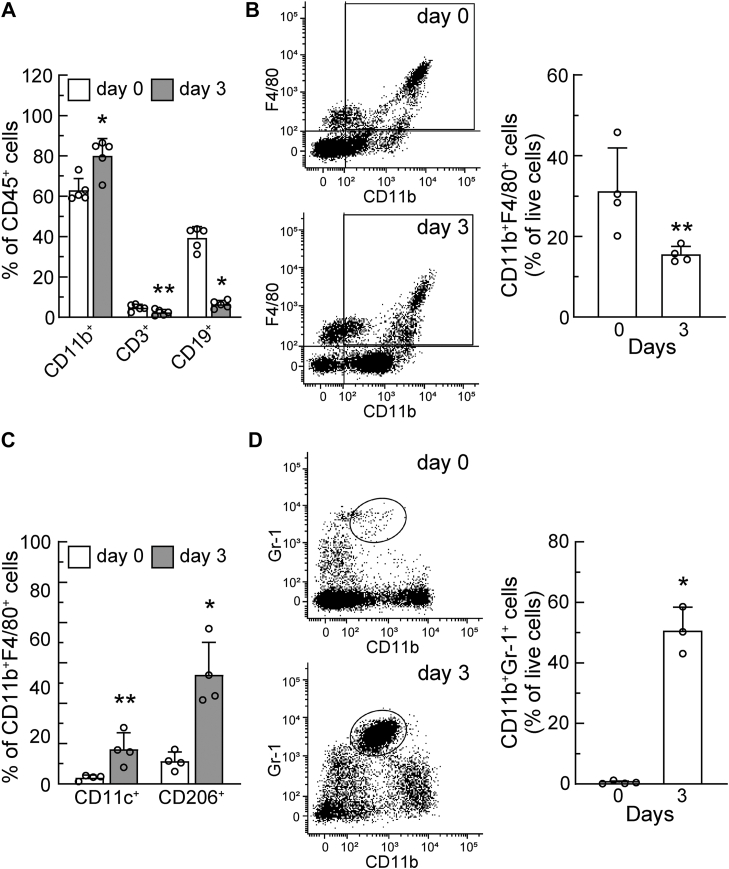
**Macrophages and myeloid-derived suppressor cells were present in peritoneal exudate cells from mice with ovarian cancer.** HM-1 cells were transplanted into the peritoneal cavities of female mice as in Figure 1, and PECs were collected on day 3 post-transplant (day 3). PECs collected from untreated control mice were also prepared as the control (day 0). The compositions of immune cells in PECs were examined using flow cytometry with several specific antibodies as described in the Experimental procedures. *A*, the percentage of CD45^+^ PECs was examined in cells reactive to antibodies against CD11b (myeloid lineage cells), CD3 (T-cell), and CD19 (B-cell). *B*, PECs were analyzed with anti-CD11b and anti-F4/80 antibodies (*left*), and the percentage of CD11b^+^ F4/80^+^ cells was quantified (*right*). C, CD11b^+^ F4/80^+^ cells were further analyzed with antibodies against CD11c (M1 macrophages) and CD206 (M2 macrophages), and the ratio of CD11c^+^ or CD206^+^ to CD11b^+^ F4/80^+^ cells was quantified. D, PECs were analyzed with anti-CD11b and anti-Gr-1 antibodies (*left*), and the percentage of CD11b^+^Gr-1^+^ cells was quantified (*right*). ∗*p* < 0.01, ∗∗*p* < 0.05 *versus* day 0.

We next focused on PECs and their cellular composition. We examined the population of HM-1 cells present in PECs from tumor-bearing mice. To specifically detect HM-1 cells in PECs, HM-1-GFP cells were transplanted into the peritoneal cavities of female mice, as described in the Experimental procedures. PECs were collected on day 3 after HM-1-GFP cell transplantation, and GFP-positive cells were analyzed by flow cytometry. The percentage of HM-1-GFP cells in the PEC fraction was 2.4 ± 0.37%, suggesting that tumor cells accounted for a small percentage of PECs on day 3 after transplantation. To detect immune cells, PECs were analyzed using the anti-CD45 antibody (the leukocyte common antigen) (38). The percentages of CD45^+^ cells in PECs from HM-1 cell-transplanted mice (day 3) and those from control (day 0) mice were 97.4 ± 1.6 and 92.2 ± 2.8%, respectively. These results indicate that leukocyte lineage immune cells were mainly present in PECs from mice with and without the transplantation of HM-1 cells. To further assess the cell populations in PECs, CD45^+^ PECs were examined by assessing reactivities to antibodies against CD11b (myeloid lineage), CD3 (T-cell lineage), and CD19 (B-cell lineage). In Figure 5*A* the percentage of CD11b^+^ cells in CD45^+^ PECs was approximately 60% in control mice (day 0), but increased to 80% in HM-1 cell-transplanted mice (day 3). The percentage of CD3^+^ cells in CD45^+^ PECs was lower than myeloid or B-cell lineage cells (day 0), and was also significantly lower in mice with ovarian cancer (day 3). CD19^+^ cells accounted for approximately 40% of CD45^+^ PECs in control mice (day 0), and this significantly decreased to 10% in mice with ovarian cancer (day 3). Collectively, the percentage of myeloid lineage cells to total leukocyte cells in PECs was significantly increased by ovarian tumor transplantation (day 3); however, myeloid lineage cells were initially the main population in PECs from untreated control mice (day 0).

Tumor-associated macrophages are reportedly abundant in tumor tissues and ascites, and are of clinical relevance in ovarian cancer (39, 40). Therefore, we focused on macrophages in PECs. In Figure 5*B*, PECs were examined by flow cytometry using antibodies against CD11b and F4/80 (macrophage) (41). The results obtained showed that the percentages of CD11b^+^F4/80^+^ PECs in transplanted mice (day 3) and controls (day 0) were 15.5 ± 2.0 and 31.2 ± 10.7%, respectively. Since the total number of PECs markedly increased 3 days after the transplantation of HM-1 cells (from 1.73 × 10^6^ ± 0.62 × 10^6^ cells/mouse to 4.86 × 10^7^ ± 1.26 × 10^7^ cells/mouse, Fig. 2*C*), the number of macrophages in the peritoneal cavity also increased after HM-1 cell transplantation. CD11b^+^F4/80^+^ PECs were examined using antibodies against CD11c (M1 macrophage) and CD206 (M2 macrophage). The results shown in Figure 5*C* demonstrated that among CD11b^+^F4/80^+^ PECs, the ratio of CD11c^+^ or CD206^+^ to CD11b^+^F4/80^+^ was significantly higher in HM-1 cell-transplanted mice (day 3) than in the controls (day 0). In addition, the ratio of CD206^+^ to CD11b^+^F4/80^+^ appeared to be higher than that of CD11c^+^ to CD11b^+^F4/80^+^; however, both ratios were increased by HM-1 cell transplantation.

In myeloid lineage cells, myeloid-derived suppressor cell (MDSCs) may also be present in PECs from HM-1 cell-transplanted mice. Cui *et al.* initially reported the involvement of MDSCs in the tumor immunity of ovarian cancer (42, 43). To investigate whether MDSCs are involved in tumor-induced intraperitoneal cells, PECs were prepared from HM-1 cell-transplanted mice or the controls and examined by flow cytometry using antibodies against CD11b and Gr-1 (MDSCs) (44) (Fig. 5*D*). The results obtained showed that the percentages of CD11b^+^Gr-1^+^ PECs from transplanted mice (day 3) and the controls (day 0) were 50.7 ± 7.8 and 0.5 ± 0.5%, respectively. These results indicated that the population of MDSCs markedly increased in PECs from mice with ovarian cancer, but was low in control cells (day 0).

### *C*-Man-Trp levels were higher in macrophages than in MDSCs from PECs of mice with ovarian cancer

**Figure 6:**
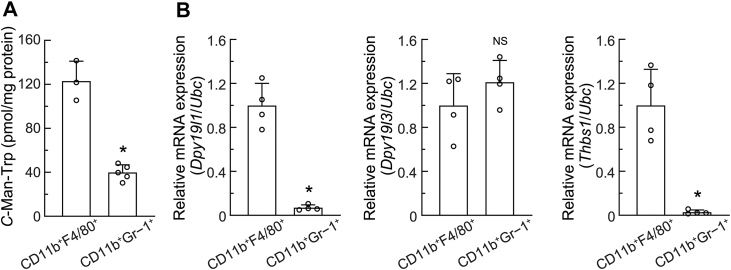
***C*-Man-Trp levels and *C*-Man-Trp metabolism-related gene expression were both higher in macrophages than in myeloid-derived suppressor cells from peritoneal exudate cells of mice with ovarian cancer.** HM-1 cells were transplanted into the peritoneal cavities of female mice as in Figure 1, and PECs were collected on day 3 post-transplant. Fractions of macrophages (CD11b^+^F4/80^+^) and MDSCs (CD11b^+^Gr-1^+^) were isolated from PECs using a cell sorter as described in the Experimental procedures. *A*, *C*-Man-Trp levels were quantified in cell fractions of CD11b^+^F4/80^+^ and CD11b^+^Gr-1^+^. B, *Dpy19l1*, *Dpy19l3*, and *Thbs1* mRNA levels were estimated by RT-qPCR in cell fractions of CD11b^+^F4/80^+^ and CD11b^+^Gr-1^+^. *Ubc* mRNA was used as the reference for the normalization. ∗*p* < 0.01, NS; not significant *versus* CD11b^+^F4/80^+^. MDSC, myeloid-derived suppressor cell; RT-qPCR, reverse-transcription quantitative PCR; *Ubc*, ubiquitin.

To identify the cells in PECs that contribute to *C*-Man-Trp production, fractions of macrophages (CD11b^+^F4/80^+^) and MDSCs (CD11b^+^Gr-1^+^) were isolated from PECs collected from HM-1 cell-transplanted mice using a cell sorter. As shown in Figure 6*A*, *C*-Man-Trp levels in macrophages (CD11b^+^F4/80^+^) and MDSCs (CD11b^+^Gr-1^+^) were quantified using an ultra-performance liquid chromatography assay. The results obtained revealed that *C*-Man-Trp levels were significantly higher in macrophages (CD11b^+^F4/80^+^) than in MDSCs (CD11b^+^Gr-1^+^). In Figure 6*B*, RNA samples were prepared from cellular fractions of macrophages (CD11b^+^F4/80^+^) and MDSCs (CD11b^+^Gr-1^+^), and *Dpy19l1*, *Dpy19l3*, and *Thbs1* mRNA levels were then assessed by RT-qPCR. *Dpy19l1* and *Thbs1* mRNA levels were both significantly higher in macrophages (CD11b^+^F4/80^+^) than in MDSCs (CD11b^+^Gr-1^+^). In contrast, *Dpy19l3* mRNA levels were similar in macrophages and MDSCs. Collectively, these results indicate that macrophages play a dominant role in *C*-Man-Trp metabolism and dynamics in the PECs of HM-1 cell-transplanted mice.

### PECs-derived macrophages from mice with ovarian cancer produced C-Man-Trp under *ex vivo* culture conditions

**Figure 7:**
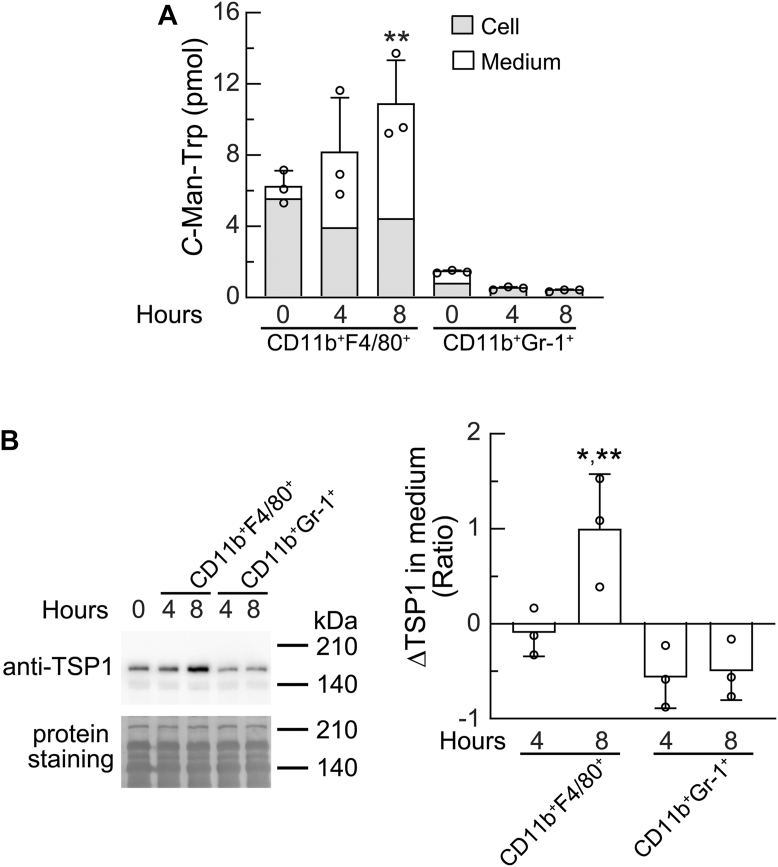
***C*-Man-Trp production in *ex vivo*-cultured macrophages and myeloid-derived suppressor cells from peritoneal exudate cells of mice with ovarian cancer.** HM-1 cells were transplanted into the peritoneal cavities of female mice as in Figure 1, and PECs were collected 3 days later. Macrophages and MDSCs were isolated from PECs using a fluorescence-activated cell sorter with several specific antibodies as described in the Experimental procedures. *A*, isolated macrophages (CD11b^+^F4/80^+^) and MDSCs (CD11b^+^Gr-1^+^) were cultured *ex vivo* for 8 h as described in the Experimental procedures. *C*-Man-Trp levels were measured in the cell (Cell) and conditioned medium (*Medium*) at the indicated time points. ∗∗*p* < 0.05 *versus* Time (0 h). *B*, the level of TSP1 secreted into the conditioned medium was examined using an immunoblot analysis at the indicated time points. Band intensity of TSP1 was quantified as described in the Experimental procedures. The change of TSP1 level in medium (ΔTSP1) was calculated based on the TSP1 level in preconditioned medium as described in the Experimental procedures. ∗*p* < 0.01 *versus* CD11b^+^GR-1^+^ (4 and 8 h). ∗∗*p* < 0.05 *versus* CD11b^+^F4/80^+^ (4 h). MDSC, myeloid-derived suppressor cell; TSP1, thrombospondin 1.

To further examine whether PECs-derived macrophages contribute to the production of *C*-Man-Trp, fractions of macrophages and MDSCs were isolated from the PECs of HM-1 cell-transplanted mice as shown in Figure 6, and were subjected to an *ex vivo* culture as described in the Experimental procedures. To measure *C*-Man-Trp levels, cell and conditioned medium samples were taken from the culture after a 4- or 8-h incubation period. In PECs-derived macrophages (CD11b^+^F4/80^+^), the cellular level of *C*-Man-Trp showed little change, whereas its level in the medium gradually increased (Fig. 7*A*). The total amount of *C*-Man-Trp was significantly higher after an 8-h *ex vivo* culture than at 0 h. In contrast, in PECs-derived MDSCs (CD11b^+^Gr-1^+^), up-regulated production of *C*-Man-Trp was not observed after an 8-h *ex vivo* culture (Fig. 7*A*). These results indicate that *C*-Man-Trp was produced by PECs-derived macrophages under *ex vivo* culture conditions. As shown in Figure 6, the expression of *Thbs1* was significantly high in PECs-derived macrophages from mice with ovarian cancer. Therefore, we examined the level of TSP1 secreted in the conditioned medium using an immunoblot analysis. As shown in Figure 7*B*, the level of secreted TSP1 was significantly elevated in PECs-derived macrophages (CD11b^+^F4/80^+^) but not in PECs-derived MDSCs (CD11b^+^Gr-1^+^) after 8 h of *ex vivo* culture. These results suggest the involvement of *C*-mannosylated proteins, such as TSP1, in the production of *C*-Man-Trp by PECs-derived macrophages from mice with ovarian cancer.

### Macrophage depletion decreased C-Man-Trp production in PECs from mice with ovarian cancer

**Figure 8:**
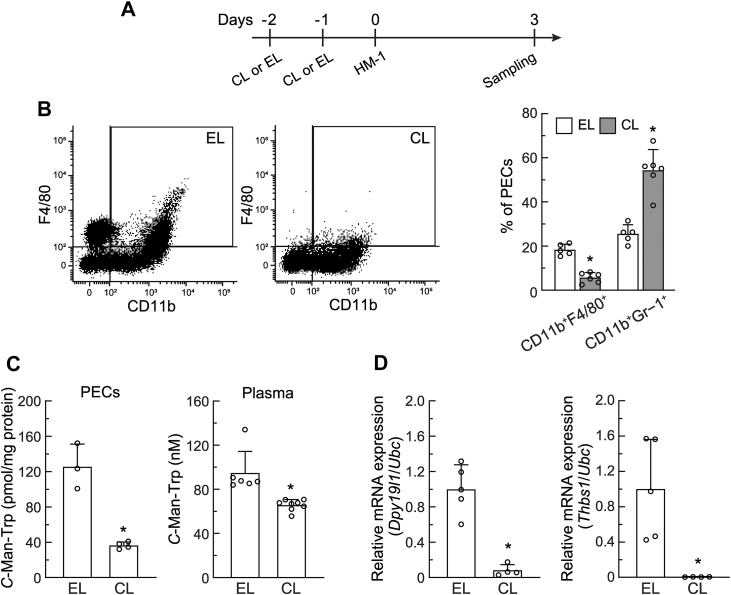
**Early-phase effects of macrophage depletion on *C*-Man-Trp levels and *C*-Man-Trp metabolism-related gene expression in mice with ovarian cancer.***A*, female mice were intravenously injected with CL or EL, and HM-1 cells were transplanted into the peritoneal cavities of mice. Mice were sacrificed 3 days after transplantation, and blood, urine, and PECs were collected for examination. *B*, compositions of macrophages in PECs from mice treated with CL or EL were examined using flow cytometry with several specific antibodies (CD11b^+^, F4/80^+^, and Gr-1^+^). The relative levels of macrophages and MDSCs are shown in the graph. *C*, *C*-Man-Trp levels in PEC and plasma samples were quantified. D, *Dpy19l1* and *Thbs1* mRNA levels were estimated by RT-qPCR in PECs from mice treated with CL or EL. *Ubc* mRNA was used as the reference for the normalization. ∗*p* < 0.01 *versus* EL. MDSC, myeloid-derived suppressor cell; RT-qPCR, reverse-transcription quantitative PCR; CL, clodronate liposomes; EL, empty liposomes; *Ubc*, ubiquitin.

The number of PECs peaked on day 3 after HM-1 cell transplantation (Fig. 2*C*). To investigate the early effects of macrophage depletion on *C*-Man-Trp and *C*-Man-Trp metabolism-related molecules in PECs from mice with ovarian cancer, female mice were intravenously injected with clodronate liposomes (CL) or empty liposomes (EL) twice at a 24-h interval. HM-1 cells were then transplanted into the peritoneal cavities of mice, and biological samples (*i.e.*, plasma, PECs, and urine) were collected and tested 3 days later, as shown in Figure 8*A*. In Figure 8*B* left, PECs were prepared from HM-1 cell-transplanted mice with the intravenous injection of CL or EL, and were examined by a flow cytometric analysis using the antibodies against CD11b and F4/80. The results obtained showed that the macrophage fraction (CD11b^+^F4/80^+^) appeared to be reduced in PECs from HM-1 cell-transplanted mice treated with CL. A significant decrease in macrophages was confirmed by quantification data (Fig. 8*B* right). The MDSC fraction (CD11b^+^Gr-1^+^) increased in PECs from HM-1 cell-transplanted mice treated with CL. As shown in Figure 8*C*, *C*-Man-Trp levels were significantly lower in both PECs (left) and plasma (right) from HM-1 cell-transplanted mice treated with CL than from those injected with EL. In addition, the level of *C*-Man-Trp excreted in urine was significantly lower in HM-1 cell-transplanted mice treated with CL (Fig. S1*A*). In Figs. 8*D* and S1*B*, transcriptional levels of *Dpy19l1*, *Dpy19l3*, and *Thbs1* in total RNA prepared from PECs were estimated by RT-qPCR. The results obtained indicated that the expression of all genes related to *C*-Man-Trp metabolism was significantly lower in PECs from HM-1 cell-transplanted mice treated with CL than in those from mice injected with EL.

### Macrophage depletion decreased C-Man-Trp production in the ascites and tumor masses of mice with ovarian cancer

**Figure 9:**
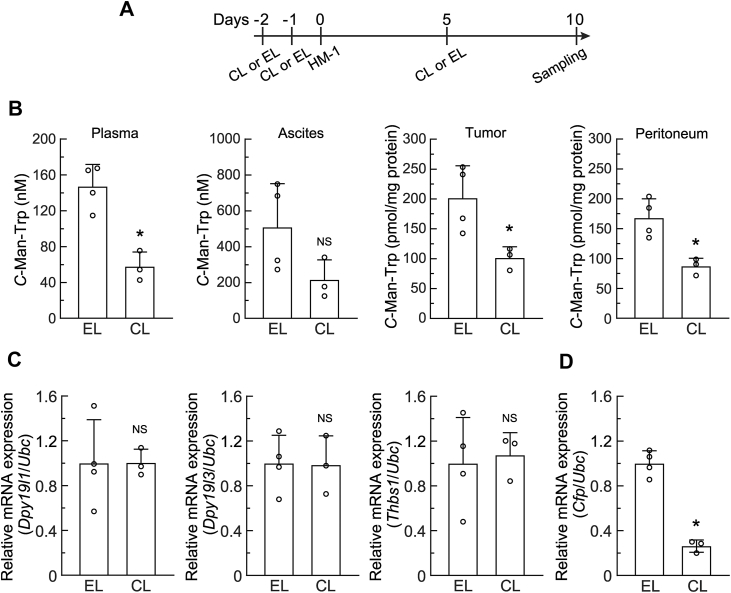
**Late-phase effects of macrophage depletion on *C*-Man-Trp levels and *C*-Man-Trp metabolism-related gene expression in mice with ovarian cancer.***A*, female mice were intravenously injected with CL or empty liposomes (EL), and HM-1 cells were then transplanted into the peritoneal cavities of mice. *B*, after transplantation, mice was sacrificed on day 10, and blood, ascites, tumor masses, and peritoneal tissue samples were collected for examination. *C*-Man-Trp levels were quantified in plasma, ascites, tumor mass, and peritoneal tissue samples. *C*, *Dpy19l1*, *Dpy19l3*, and *Thbs1* mRNA levels were estimated by RT-qPCR in tumor masses from mice treated with CL or EL. *D*, *Cfp* mRNA levels in tumor masses from mice treated with CL or EL were estimated by RT-qPCR. *Ubc* mRNA was used as the reference for the normalization. ∗*p* < 0.01, NS, not significant *versus* EL. RT-qPCR, reverse-transcription quantitative PCR; CL, clodronate liposomes; EL, empty liposomes; *CFP, complement factor properdin; Ubc,* ubiquitin.

Tumor masses were detected 7 days after the transplantation of HM-1 cells, and *C*-Man-Trp levels in tumor masses peaked on day 10 after transplantation (Fig. 1*B*). Therefore, to investigate the late effects of macrophage depletion on *C*-Man-Trp and *C*-Man-Trp metabolism-related molecules, female mice were intravenously injected with CL or EL, and HM-1 cells were then transplanted into their peritoneal cavities. Biological samples (*i.e.*, plasma, ascites, tumor masses, and peritoneal tissues) were collected 10 days later and tested, as shown in Figure 9*A*. In Figure 9*B*, *C*-Man-Trp levels were quantified in several biological samples, and were significantly lower in plasma, ascites, tumor masses, and peritoneal tissues from HM-1 cell-transplanted mice treated with CL than from those injected with EL. The transcriptional levels of *Dpy19l1*, *Dpy19l3*, and *Thbs1* in total RNA prepared from tumor masses (Fig. 9*C*) and peritoneal tissues (Fig. S2) were examined by RT-qPCR. No significant differences were observed in gene expression in tumor masses or peritoneal tissues between the treatments with CL and EL. We also investigated complement factor properdin (CFP) because it is a plasma *C*-mannosylated protein containing at least 14 *C*-mannosylation consensus sites (45, 46) and its production is mostly limited in leukocytes containing macrophages (47). In Figure 9*D*, the transcriptional level of *Cfp* in total RNA prepared from tumor masses was examined by RT-qPCR. The results obtained indicate that *Cfp* mRNA expression was significantly lower in tumor masses from HM-1 cell-transplanted mice treated with CL than in those from mice injected with EL.

### Macrophage depletion decreased C-Man-Trp production in peritoneal cavity cells from normal healthy mice

**Figure 10:**
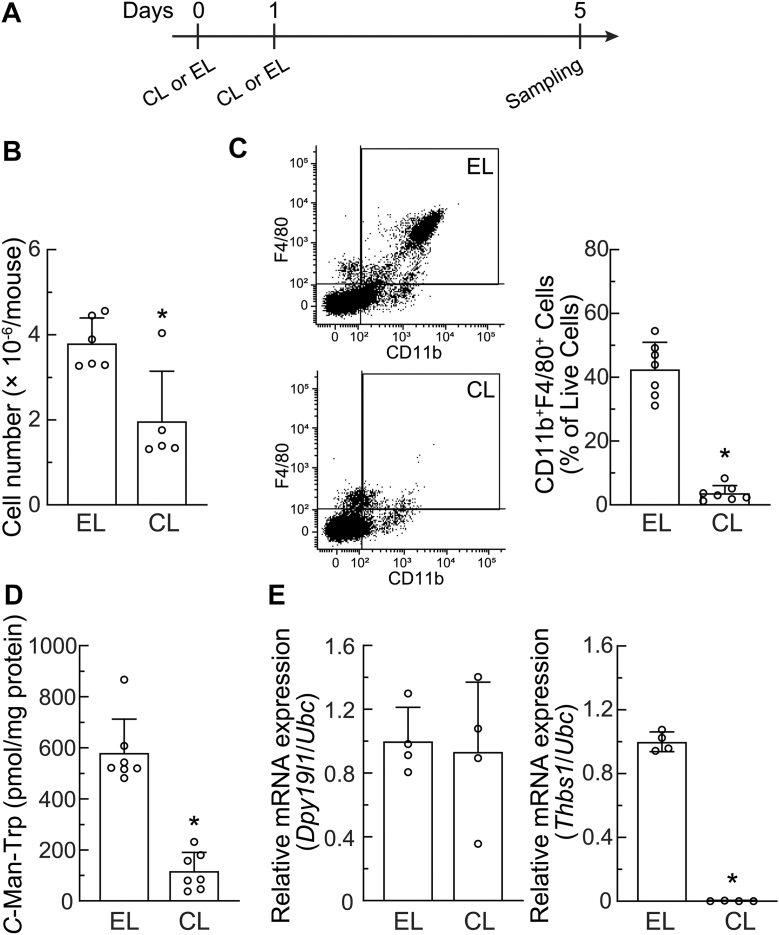
**Effects of macrophage depletion on *C*-Man-Trp levels and *C*-Man-Trp metabolism-related gene expression in peritoneal cavity cells of normal healthy mice.***A*, female mice were intravenously injected with CL or empty liposomes (EL). Mice were sacrificed 5 days later, and peritoneal cavity cells were collected for analyses. *B*, the number of peritoneal cavity cells was counted and shown. *C*, the compositions of macrophages in peritoneal cavity cells were examined using flow cytometry with specific antibodies (CD11b^+^, F4/80^+^) (*left*). The percentage of CD11b^+^F4/80^+^ cells was quantified (*right*). *D*, *C*-Man-Trp levels were quantified in peritoneal cavity cells. E, *Dpy19l1* (*left*) and *Thbs1* (*right*) mRNA levels in peritoneal cavity cells were estimated by RT-qPCR. ∗*p* < 0.01 *versus* EL. RT-qPCR, reverse-transcription quantitative PCR; CL, clodronate liposomes; EL, empty liposomes.

To investigate whether macrophages contribute to *C*-Man-Trp production in the bodies of normal mice without ovarian tumors, female mice were intravenously injected with CL or EL, and peritoneal cavity cells were collected 5 days later and tested as shown in Figure 10*A*. In Figure 10*B*, the number of peritoneal cavity cells was significantly lower in normal mice treated with CL than in those treated with EL. No ascites was observed in mice treated with CL or EL. In Figure 10*C* left, the macrophage fraction (CD11b^+^F4/80^+^) appeared to be reduced in the peritoneal cavity cells of mice with CL. The CL-induced decrease in macrophages was confirmed by quantification data (Fig. 10*C* right). In Figure 10*D*, *C*-Man-Trp levels were significantly lower in the peritoneal cavity cells of mice treated with CL. *Thbs1* mRNA levels were significantly lower in the peritoneal cavity cells of mice with CL (Fig. 10*E* right), while no significant difference was observed in *Dpy19l1* levels between the CL and EL treatments (Fig. 10*E* left). The transcriptional expression of *Dpy19l3* appeared to be slightly up-regulated in mice treated with CL; however, no significant differences were observed between the CL and EL treatments (Fig. S3).

### Macrophage depletion decreased C-Man-Trp production in the plasma and liver of normal healthy mice

**Figure 11:**
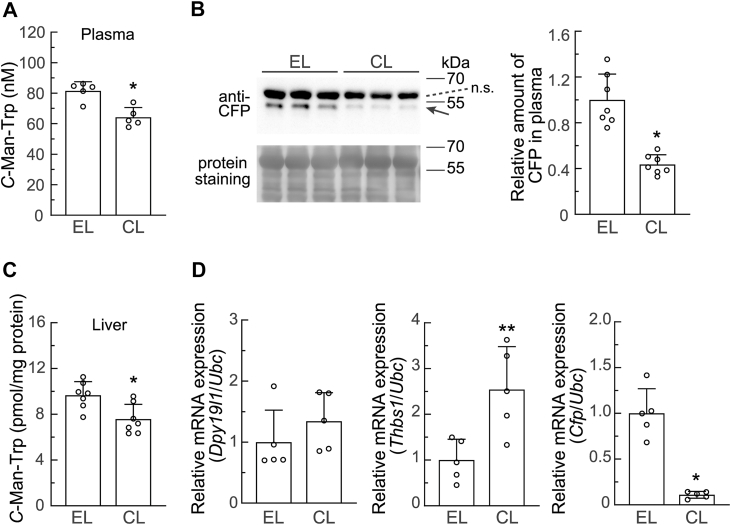
**Effects of macrophage depletion on *C*-Man-Trp levels and the expression of *C*-Man-Trp metabolism-related molecules in plasma and liver tissues from normal healthy mice.** Female mice were intravenously injected with CL or EL. Mice were sacrificed 5 days later, and plasma and liver tissues were collected for analyses. *A*, *C*-Man-Trp levels were quantified in plasma. *B*, CFP levels in plasma was examined using an immunoblot analysis with the antibody against CFP. Total proteins on the membranes were stained as described in the Experimental procedures, and used for the loading controls of plasma. Band intensity of CFP was quantified as described in the Experimental procedures. n.s., non-specific. *C*, *C*-Man-Trp levels were quantified in liver tissue samples. *D*, *Dpy19l1* (*left*), *Thbs1* (*middle*), and *Cfp* (*right*) mRNA levels in liver tissue samples were estimated by RT-qPCR. ∗*p* < 0.01, ∗∗*p* < 0.05 *versus* EL. RT-qPCR, reverse-transcription quantitative PCR; CL, clodronate liposomes; EL, empty liposomes; *CFP, complement factor properdin*.

*C*-Man-Trp levels in plasma were also examined in normal healthy mice treated with CL or EL (Fig. 10*A*), and were significantly lower in normal mice treated with CL than in those treated with EL (Fig. 11*A*). To obtain insights into the mechanisms underlying the decrease in *C*-Man-Trp levels due to macrophage depletion, we focused on CFP. In Figure 11*B*, CFP levels in the plasma of normal mice treated with CL or EL were assessed using an immunoblot analysis. The CFP band (arrow) was clearly detected in the plasma of mice treated with EL, but was weaker in the plasma of those treated with CL. Quantitative data also showed that CFP levels in plasma were significantly lower in mice treated with CL than in those treated with EL (Fig. 11*B* right).

The effects of macrophage depletion on *C*-Man-Trp metabolism-related molecules were also investigated in the liver because tissue-resident macrophages (*i.e.*, Kupffer cells) are abundant in liver tissues (48). *C*-Man-Trp levels in liver tissues were significantly lower in mice treated with CL than in those treated with EL (Fig. 11*C*). Regarding the transcriptional expressions of *Dpy19l1*, *Thbs1*, and *Cfp* in liver tissues, *Thbs1* mRNA levels were significantly higher in mice treated with CL than in those treated with EL (Fig. 11*D* middle), whereas *Dpy19l1* mRNA levels were not (Fig. 11*D* left). The transcriptional expression of *Dpy19l3* appeared to be slightly higher in mice treated with CL than in those treated with EL, while no significant differences were observed in its levels (Fig. S4). In contrast, the expression of *Cfp* mRNA was significantly lower in mice treated with CL than in those treated with EL (Fig. 11*D* right).

Collectively, these results showed that *C*-Man-Trp levels were significantly lower in the peritoneal cavity cells (Fig. 10*C*), plasma (Fig. 11*A*), and liver tissues (Fig. 11*C*) of normal healthy mice treated with CL than in those of mice treated with EL.

## Discussion

Ovarian cancer is a gynecologic cancer with a high mortality rate among female malignant tumors (49, 50, 51). Regarding glycosylation-related molecules in ovarian cancer, previous studies reported characteristic changes in the expression of glycoproteins (*e.g.*, MUC16, MUC1, MUC20, AFP, CEA, and HE4) (52) in these patients. We also demonstrated that *C*-Man-Trp levels in plasma were elevated in patients with ovarian tumors, with further increases being observed in malignant cases (24). Collectively, these findings suggest the involvement of changes in protein glycosylation in the pathophysiology of ovarian cancer. Increases in the plasma level of *C*-Man-Trp may be a novel change in glycosylation metabolism associated with the pathophysiology of ovarian cancer.

The molecular mechanisms responsible for increases in *C*-Man-Trp in the tissues and body fluids of mammals with ovarian cancer remain unclear. We previously reported that autophagy induced an increase in *C*-Man-Trp in cultured cells (8). The production of *C*-Man-Trp was also observed when mouse liver-derived lysosomal fractions were incubated with human platelet-derived TSP1, a *C*-mannosylated protein (53), suggesting that *C*-Man-Trp was produced *via* the lysosomal degradation of *C-*mannosylated proteins. Increases in the production of *C*-Man-Trp may be regulated as follows: (I) an increase in the *C*-mannosylation of substrate proteins, (II) an increase in the expression of substrate proteins for *C*-mannosylation, and (III) an increase in and/or the activation of the degrading system related to *C*-Man-Trp production. Regarding human cancers, the mRNAs of *C*-mannosyltransferases (*DPY19L1* and *DPY19L3*) have been detected in all cancer tissues and exhibit low cancer specificity based on The Cancer Genome Atlas datasets (The Human Protein Atlas). In the present study, *Dpy19l1* and *Dpy19l3* mRNA levels significantly increased in PECs and peritoneal tissues after tumor transplantation (Fig. 3). These results indicate that the expression of protein *C*-mannosyltransferases contributed in part to the increased production of *C*-Man-Trp in mice with ovarian tumors. Although the regulation of *DPY19L1* and *DPY19L3* expression has not yet been investigated, further studies on the mechanisms underlying the tumor-stimulated induction of both genes are required to obtain a more detailed understanding of increases in the production of *C*-Man-Trp in ovarian cancer.

More than 100 proteins have been reported to be *C*-mannosylated (25, 54, 55, 56), most of which are secretory or membrane proteins, and approximately half of these proteins are TSR superfamily proteins. In the present study, we examined the expression levels of TSR superfamily proteins related to ovarian cancer. Pathophysiological relationships to ovarian cancer have been reported for TSR superfamily proteins, such as TSP1 (32, 33), spondin 1 (34, 57), and CCN1 (36). TSP1 is an extracellular protein with various biomedical functions related to angiogenesis, apoptosis, autophagy, cell proliferation, and tumor biology (58, 59, 60). Spondin 1 was initially identified as a functional protein involved in neural development (61), and previous studies showed increases in its mRNA (*Spon1*) in ovarian tumor tissues from humans (34, 35, 62, 63). In the protein expression summary in the PAN-CANCER COHORT (The Human Protein Atlas), the expression of spondin 1 was highly up-regulated in the blood of ovarian cancer and myeloma patients. As a matricellular protein, CCN1 has diverse cellular functions related to cell adhesion, migration, proliferation, and apoptosis (64). Aberrant CCN1 expression has been associated with the development and progression of cancers in a number of tissues, including the ovary (65). Although *C*-mannosylation of CCN1 has not been reported, its secretion was suppressed by *DPY19L3* knockout in human induced pluripotent stem cells (66), suggesting that CCN1 was *C*-mannosylated and that this might function in its secretion. Similar effects of *C*-mannosylation on protein secretion have been reported for various TSR superfamily proteins (25, 66). In the present study, *Thbs1* and *Spon1* mRNA levels were increased in tumor masses, peritoneal tissues with disseminated sites, and PECs. In addition, *Ccn1* mRNA was up-regulated in peritoneal tissues with disseminated sites and PECs. Therefore, TSP1, spondin 1, and CCN1 may be involved in pathophysiological processes in ovarian cancer dissemination, and may also contribute to the modified dynamics of *C*-Man-Trp in the ovarian tumor-induced microenvironment by providing some sources of *C*-Man-Trp.

In this murine ovarian cancer metastasis model, we attempted to identify the cells responsible for the increased production of *C*-Man-Trp. We focused on the composition of PECs, which contained a large amount of *C*-Man-Trp. In Figure 5, PECs contained a large fraction of myeloid lineage cells (CD11b^+^) accounting for approximately 80% of all leukocyte lineage cells. The myeloid fraction mainly consisted of MDSCs (CD11b^+^Gr-1^+^, 50%) and macrophages (CD11b^+^F4/80^+^, 15%). In addition, the M2 fraction (CD206^+^) (67) was markedly larger, while the M1 fraction (CD11c^+^) (68) was larger in the macrophage fraction from mice with ovarian cancer than in that from control mice. These results are consistent with previous findings on tumor-associated dynamics in immune cells through the progression of ovarian cancer (39, 40, 42, 43, 69). On the other hand, it is important to note that the PECs of mice with ovarian cancer contained a small fraction of HM-1 tumor cells (<3%). In addition, *C*-Man-Trp levels were low in HM-1 cells (Fig. 1*B*). These results suggest the negligible contribution of HM-1 cells to the production of *C*-Man-Trp in PECs from mice with ovarian cancer. However, *C*-Man-Trp metabolism in HM-1 cells may also have been involved to some extent in the increased production of *C*-Man-Trp. Further studies are warranted to address the functional role of HM-1 cells in the increased production of *C*-Man-Trp in mice transplanted with HM-1 cells. The cellular level of *C*-Man-Trp and *Dpy19l1* and *Thbs1* mRNA levels were significantly higher in macrophages than in MDSCs (Fig. 6), suggesting that macrophages play a dominant role in the up-regulation of *C*-Man-Trp.

Under *ex vivo* culture conditions, the total amount of *C-*Man-Trp significantly increased in PECs-derived macrophages and the conditioned medium after an 8-h culture (Fig. 7*A*), indicating that *C*-Man-Trp was produced in the presence of PECs-derived macrophages. The greater increase in *C*-Man-Trp in ascites than in plasma (Fig. 1*F*) may be due to *C*-Man-Trp being released from PECs-derived macrophages in addition to the degradation of *C-*mannosylated proteins in ascites. TSP1 was detected and its levels increased in the *ex vivo* culture medium (Fig. 7*B*). TSP1 was also present in the plasma and ascites of mice with ovarian cancer (Fig. 4), with lower levels being observed in the latter than in the former. On the other hand, after tumor transplantation, *Thbs1* mRNA levels markedly increased, whereas the cellular content of TSP1 significantly decreased in PECs. Collectively, these results suggest that in the peritoneal cavity of mice with ovarian cancer, PECs-derived macrophages may produce and secrete high levels of TSP1. In addition, increases in the degradation of TSP1 in ascites may promote the production of *C-*Man-Trp in mice with ovarian cancer.

Upon the pretreatment of mice with CL, the number of macrophages in the PEC fraction was significantly lower, and the cellular level of *C*-Man-Trp was also lower in PECs (on day 3 after transplantation) and tumor masses (on day 10 after transplantation) than in those from mice pretreated with EL (Figs. 8 and 9). The transcriptional levels of *Dpy19l1*, *Dpy19l3*, and *Thbs1* were significantly lower in PECs from HM-1 cell-transplanted mice treated with CL than in those from mice injected with EL (Figs. 8*D* and S1*B*). These results suggest that macrophages are mainly involved in the production and degradation of *C*-mannosylated proteins leading to *C*-Man-Trp production in PECs. In contrast, *Dpy19l1*, *Dpy19l3*, and *Thbs1* levels in tumor masses or peritoneal tissues did not significantly differ between the CL and EL treatments (Figs. 9*C* and S2). These results suggest the involvement of not only macrophages, but also various tumor microenvironment-associated cells (70) in the dynamics of *C*-Man-Trp production in tumor masses. As shown in Figure 9*D*, *Cfp* mRNA expression was significantly lower in tumor masses from HM-1 cell-transplanted mice treated with CL than in those from mice injected with EL. Therefore, macrophages in tumor masses are considered to contribute to *C*-Man-Trp production by providing the *C*-mannosylated substrate CFP, however, they may also produce *C*-Man-Trp by degrading *C*-mannosylated proteins derived from other tumor-associated cells (70). Further investigations are required to clarify how macrophages function for *C*-Man-Trp dynamics in the ovarian tumor microenvironment.

These results strongly suggest that at least some *C*-Man-Trp in mice with ovarian cancer was derived from tumor-stimulated macrophages following the up-regulated expression and enhanced proteolysis of *C*-mannosylated proteins, such as TSP1 and CFP. TSP1 binds to macrophage receptors (*e.g.*, CD36 and CD47) for cell signaling related to anti-angiogenesis, tumor immunity, or apoptosis (71). Therefore, CD36 and CD47 on the cell surface of macrophages may contribute to the uptake and degradation turnover of TSP1, and may result in an increase in *C*-Man-Trp production in tumor-stimulated macrophages. In addition, various tumor microenvironment-associated cells other than macrophages may be involved in providing *C*-mannosylated proteins as a source for *C*-Man-Trp production in ovarian cancer. The TSP1 protein is reportedly degraded by various proteases related to blood coagulation and fibrinolysis (*e.g.*, thrombin and plasmin) and leukocyte inflammation (*e.g.*, cathepsin and elastase) (72), or by kallikrein-related peptidase seven secreted from ovarian cancer cells *in vitro* (73). These studies suggest the involvement of a number of proteases/peptidases in the proteolytic degradation of *C*-mannosylated proteins outside of cells (in ascites and plasma) and *C*-Man-Trp production. Therefore, the identification of proteases/peptidases involved in *C*-Man-Trp production in the peritoneal cavities of mice with ovarian tumors is a challenge for the future.

In the present study, we also examined the effects of macrophage depletion with CL on the levels of *C*-Man-Trp and *C*-Man-Trp metabolism-related molecules in selected tissue samples from normal healthy mice (Figs. 10 and 11). Macrophage depletion caused a decrease in the basal levels of *C*-Man-Trp in peritoneal cavity cells, plasma, and liver. These results suggest the involvement of macrophages in the maintenance of *C-*Man-Trp homeostasis, even in normal healthy mice. The CL-induced down-regulation of *C*-Man-Trp in the plasma and peritoneal cavity cells of healthy mice appeared to be similar to its down-regulation in those from mice with ovarian cancer. Regarding *C*-Man-Trp metabolism-related molecules, *Thbs1* mRNA levels in peritoneal cavity cells were significantly decreased by CL in mice with ovarian cancer and normal healthy mice (Figs. 8*D* and 10*E*). On the other hand, *Dpy19l1* mRNA levels were decreased by CL in the PECs of mice with ovarian cancer, but not in the peritoneal cavity cells of healthy mice (Figs. 8, *D* and 10*E*). The dynamics of *C*-Man-Trp production may involve different regulatory mechanisms in unstressed peritoneal cavity cells and tumor-stimulated PECs. Furthermore, the peritoneal cavity microenvironment may markedly differ between mice with ovarian cancer and normal healthy mice, which may have an impact on the balance between the influx and efflux of small molecules, such as *C-*Man-Trp in various cells constituting PECs. Further research with a focus on the microenvironment surrounding PECs may be a forthcoming challenge.

*C-*Man-Trp levels in liver tissues were lower in normal healthy mice treated with CL than in those injected with EL (Fig. 11*C*). This result may be attributed to the elimination of a large number of macrophages (*i.e.*, Kupffer cells) in liver tissues under the conditions with CL. As shown in the peritoneal cavity cells of healthy mice with CL, *Dpy19l1* mRNA levels in liver tissues were similar in normal healthy mice treated with EL and CL (Fig. 11*D*). On the other hand, *Thbs1* mRNA levels in liver tissues were higher in healthy mice treated with CL than in those injected with EL (Fig. 11*D*). These results suggest that hepatic cells other than macrophages are mainly involved in the expression of these genes. Macrophage depletion with CL resulted in a significant decrease in *Cfp* mRNA levels in liver tissues and decreased CFP protein levels in the plasma of normal healthy mice (Fig. 11, *B* and *D*). CFP, which is mainly produced by macrophages in liver, may be a *C*-mannosylated protein substrate that is degraded for *C*-Man-Trp production. In contrast, in bone marrow and the kidney, *C*-Man-Trp levels were not affected in normal healthy mice treated with CL (Fig. S5), which indicates that *C*-Man-Trp-producing cells other than macrophages were largely responsible for the dynamics of *C*-Man-Trp in these organs. Therefore, further studies are needed to elucidate the mechanisms regulating organ- or tissue-specific *C*-Man-Trp production and homeostasis in normal healthy mice.

This is the first study to demonstrate the pivotal role of tumor-stimulated macrophages in increases in *C*-Man-Trp production through changes in the metabolism of *C*-mannosylated proteins in the cancer microenvironment of mice with ovarian cancer. Although some *C*-Man-Trp metabolism-related molecules, such as DPY19L1, DPY19L3, TSP1, spondin 1, CCN1, and CFP, have been suggested to play a role in increases in *C*-Man-Trp production in ovarian cancer, further investigations are required to clarify the overall mechanisms regulating *C*-Man-Trp metabolism in ovarian cancer patients. This study also demonstrated the contribution of macrophages to the basal homeostasis of *C*-Man-Trp production, even in normal healthy mice, in a manner that differed from that in mice with ovarian cancer. Further study needs to be done to clarify how macrophages regulate *C*-Man-Trp metabolism. *C*-Man-Trp concentrations in blood differentially change in a number of pathological conditions other than ovarian cancer, such as kidney dysfunction, cardiovascular diseases, vascular complications, and myeloproliferative diseases. Regarding the other unexplored diseases involving *C*-Man-Trp, the role of macrophages in *C*-Man-Trp dynamics *in vivo* needs to be further investigated.

## Experimental procedures

### Materials

The PCR primers used in this study are listed in Table S1. CL and EL were purchased from Katayama Chemical (Hygieia Bioscience) (Codes 160-0429-1 and 160-0431-1). Antibodies against TSP1 (A6.1) (sc-59887) and CFP (A00852-2) were from Santa Cruz Biotechnology Inc. and BOSTER Biological Technology, respectively. Antibodies against cell surface markers used for flow cytometry analysis are following: CD11b (BD biosciences; 560456), CD11c (BioLegend; 117317); CD3 (BioLegend; PE-65077), CD19 (BD biosciences; 561739), CD45 (BioLegend; 103131), CD206, (BioLegend; 141707), F4/80 (BioRad; MCA497FA), Gr-1, (BioLegend; 108407). The other reagents or chemicals used in the present study were all high grade and obtained from Merck Sigma-Aldrich Japan Ltd or Fujifilm Wako Pure Chemical Corp.

### Cells

The mouse ovarian cancer cell line OV-2944-HM-1 (HM-1) was purchased from the Riken Bioresource Center Cell Bank. Cells were grown in Dulbecco’s modified Eagle’s medium (Glucose 4.5 g/l) supplemented with 10% fetal calf serum, 1 × GlutaMax (Life Technologies Japan Ltd), 100 U/ml penicillin, and 100 μg/ml streptomycin at 37 °C in a humidified atmosphere of 5% CO_2_/95% air. To detect HM-1 cells in peritoneal cavity cells, HM-1 cells expressing GFP (HM-1-GFP) were established. Briefly, the mammalian GFP expression plasmid pAcGFP-hygro was introduced into HM-1 cells. Cells stably expressing GFP were selected by the following steps. Cells were cultured in selective medium containing 100 μg/ml hygromycin and GFP-expressing cells were collected by a fluorescence-activated cell sorter (BD FACS Melody, BD Biosciences). Collection cycles were repeated more than 3 times, and HM-1-GFP cells were then obtained and kept in the medium with 50 μg/ml hygromycin.

### Mouse model of the peritoneal dissemination of ovarian cancer

All animal experiments were approved by the Wakayama Medical University Animal Care and Use Committee (Approved Number 1034, 1263). Female B6C3F1 mice were purchased from CLEA Japan, Inc. Mice were housed in a temperature- and light-controlled room and provided food and water ad *libitum*. HM-1 cells in the logarithmic phase were thoroughly washed with PBS and transplanted into the peritoneal cavity at 1.5 × 10^6^ cells in 1 ml of PBS/mouse. Cell viability was checked and confirmed by trypan blue dye exclusion methods (more than 97% negative).

At the time mentioned, mice were sacrificed by exposure to isoflurane, and blood and tissue samples were then harvested. Blood was transferred to tubes with K_2_EDTA (BD Microtainer, BD), and centrifuged at 1200×*g* for 15 min. The resulting plasma was transferred to a new tube and stored at −80 °C. Tissue samples were snap frozen with liquid nitrogen, and stored at −80 °C until used. To collect whole suspended peritoneal cavity cells, 9 ml of ice-cold PBS was injected into the peritoneal cavity and cells were precipitated from the suspension by centrifugation.

To analyze urine samples, mice were housed in a metabolic cage individually with free access to food and water for 24 h. Collected urine was centrifuged at 5000×*g*, and the supernatant was used to measure *C*-Man-Trp levels.

### *Ex vivo* culture of PECs-derived macrophages and MDSCs

Three days after the transplantation of HM-1 cells, mice were sacrificed. Blood samples were collected from the heart, kept at 37 °C for 30 min, and serum was prepared by centrifugation (1200×*g* at 25 °C for 20 min). PECs were collected as described above, stained cells were prepared according to the method part of “Flow cytometry”, and the CD11b^+^F4/80^+^ and CD11b^+^Gr-1^+^ populations were collected using a fluorescence-activated cell sorter (MA900, Sony Corporation). The fractions of macrophages and MDSCs in PECs were washed twice with RPMI1640 medium, and resuspended in RPMI1640 medium containing 2% serum derived from the same mouse from which PECs were collected. Isolated cells were cultured for the time described above. These cells and the conditioned medium were both harvested for the measurement of *C*-Man-Trp levels and Western blotting.

### Macrophage depletion with CL

Liposomes containing 25 μg of clodronate in a volume of 250 μl were administered intravenously twice at an interval of 24 h for macrophage depletion. Twenty-four hours after the second administration of CL, HM-1 cells were inoculated into the peritoneal cavity of mice. To examine the effects of macrophage depletion on cells infiltrating the peritoneal cavity, peritoneal lavage fluid was collected 3 days after the transplantation of HM-1 cells, as described above. To examine their effects on dissemination or tumor masses, CL were additionally administered on day 5 after the inoculation with HM-1 cells. Mice were sacrificed 10 days after the inoculation, and samples were collected as described above.

In the case of normal healthy mice, CL were also administrated intravenously twice as described above. Four days after second injection, all samples were collected. In all experiments with CL, control mice received an equivalent amount of EL.

### Measurement of *C*-Man-Trp

*C*-Man-Trp levels in samples from mice or cultured cells were analyzed as previously described with small modifications (10, 18). Briefly, *C*-Man-Trp was extracted from samples using extraction solvent (acetonitrile: methanol: formic acid = 50: 49.9: 0.1), and the cleared supernatant was analyzed with ultra-performance liquid chromatography (ACQUITY UPLC H-class system, Waters Corp.) equipped with a fluorescence detector. The separation of *C*-Man-Trp was conducted using hydrophilic interaction liquid chromatography with an ACQUITY UPLC BEH Amide column (1.7 μm, 2.1 × 100 mm, Waters Corp.). The column was kept with the initial mobile phase (10% water: 0.05% formic acid: 89.95% acetonitrile) for 1 min, and *C*-Man-Trp was developed with the second mobile phase (15% water: 0.05% formic acid: 84.95% acetonitrile) for 7 min and then detected by fluorescence (excitation at 285 nm; emission at 350 nm). *C*-Man-Trp in samples was quantified by measuring its peak area by comparisons with chemically synthesized authentic *C*-Man-Trp (74). The column was washed out with a linear gradient of 15 to 55% water: 0.05% formic acid: 84.95-44.95% acetonitrile for 5 min, held for 3 min, and then reequilibrated with the initial mobile phase for 7 min. The mobile phase was used at 0.5 ml/min and the column was kept at 40 °C.

### Renal functional analysis

In the renal functional analysis, plasma creatinine was measured using the Hitachi 7180 Clinical Analyzer (Hitachi High-Tech Corp.) with the L Type Wako CRE/M kit (creatininase-HMMPS method, Fujifilm Wako Pure Chemical Corp). This examination was conducted at the Nagahama Life Science Laboratory.

### RNA preparation and RT-qPCR

Total RNA was extracted with TRizol solution (Life Technologies Japan Ltd) according to the manufacturer’s protocol. The quantity and quality of isolated RNA were confirmed spectrophotometrically, and total RNA was reverse transcribed using the PrimeScript FAST RT reagent kit with a gDNA eraser (Takara Bio Inc.). Target DNA was amplified by Brilliant III Ultra-Fast SYBR Green QPCR Master Mixes (Agilent) with specific primers (listed in Table S1) using complementary DNA as a template, and the amplified signal was monitored by Thermal Cycler Dice Real Time System IV (Takara Bio Inc.). Expression levels of target genes were normalized to the expression level of ubiquitin gene (*Ubc*) by using the 2^−ΔΔCT^ method.

### Flow cytometry

Three days after HM-1 cell transplantation, peritoneal cavity cells were collected as previously described, and red blood cells were lysed with RBC Lysis Buffer (BioLegend). Immune cell surface Fc receptors were blocked with an anti-CD16/CD32 antibody and cells were stained with fluorescence-labeled cell lineage marker antibodies diluted in MACS buffer (1% fetal calf serum and 5 mM EDTA in PBS). After washing with PBS, dead cells were stained with the LIVE/DEAD Fixable Dead Cell Stain Kit using Aqua fluorescent reactive dye (Life Technologies Japan Ltd). Cell suspensions were filtered and analyzed using the flow cytometer BD FACSVerse (BD Biosciences).

Regarding cell fractionation, stained cells were prepared as described above, and the CD11b^+^F4/80^+^ and CD11b^+^Gr-1^+^ populations were collected using a fluorescence-activated cell sorter (MA900, Sony Corporation).

### Western blotting

PECs were lysed with RIPA buffer (0.1% SDS, 1% deoxycholate,1% NP-40, 2 mM EDTA, 150 mM NaCl, and 20 mM Tris (pH 7.6)) and the cleared supernatant was obtained with centrifugation (8000×*g* at 4 °C for 5 min). The cleared supernatant of PECs and liquid samples (conditioned culture medium of PECs, ascites, and plasma) were mixed with SDS-polyacrylamide gel electrophoresis (PAGE) loading buffer to a final concentration of 2% SDS, 60 mM Tris-HCl (pH 6.8), 40 mM DTT, 10% glycerol, and 0.012% Bromophenol Blue. Proteins were separated using SDS-PAGE and transferred to a polyvinylidene fluoride membrane (Merck Sigma-Aldrich Japan Ltd). The membranes were blocked with 5% skim milk in Tris-buffered saline containing 0.1% Tween-20 at room temperature for 1 h, and were then incubated with a primary antibody diluted in blocking solution at 4 °C overnight. The membranes were incubated with a horseradish peroxidase-conjugated secondary antibody at room temperature for 1 h. Between staining, the membranes were thoroughly washed with Tween-20. Immunostained proteins were then incubated with Immobilon Chemiluminescence HRP Substrate (Merck Sigma-Aldrich Japan Ltd) and visualized with a chemiluminescence imaging system (WSE-6100 LuminoGraph Ⅰ, ATTO Corp.). The immunostaining signals were quantified using a software CS Analyzer 4 (ATTO Corp.). Proteins on the membranes were visualized with Colloidal Gold Total Protein Stain (BioRad), and the stained bands were quantified densitometrically using ImageJ version 1.53k (National Institute of Health). The results were used to normalize their immunostaining signals to the total protein in the PECs lysate samples. Regarding TSP1 in *ex vivo* culture medium, the change of TSP1 level in medium (ΔTSP1) was assessed by the difference in TSP1-band intensity between conditioned and preconditioned media and normalized by the value for CD11b^+^F4/80^+^ (8 h).

### Statistical analysis

Data are shown as the mean ± standard deviation of at least three mice. Statistical analysis was carried out using statistical software JMP Pro (JMP Statistical Discovery LLC,). We used one-way analysis of variance (ANOVA) followed by Tukey-Kramer test or unpaired Student’s *t* test to analyze the data, with *p* values of <0.05 said to be significant.

## Data availability

All data are available in the article and supporting information files.

## Supporting information

This article contains supporting information.

## Conflict of interest

The authors declare that they have no conflicts of interest with the contents of this article.

## Supporting information

Supporting infromation
