# Supplementary material for: C-mannosyl tryptophan dynamics in a mouse model of the peritoneal dissemination of ovarian cancer
Source: J Biol Chem. 2026 Mar 9;302(5):111359. doi: 10.1016/j.jbc.2026.111359 (PMC13066745; doi:10.1016/j.jbc.2026.111359)
Supplement: Supporting infromation [file mmc1.docx]

**Supporting Information**

Title

*C*-Mannosyl tryptophan dynamics in a mouse model of the peritoneal dissemination of ovarian cancer

Authors

Yoko Inai, Shiho Minakata, Kaya Tsujimoto, Shino Manabe, Naoyuki Iwahashi, Ryota Kamijo, Yuma Nakadaira, Keisuke Nishikawa, Tomohiro Hashizume, Kazuhiko Ino, and Yoshito Ihara*

Contents

Supplementary Figure 1 (Fig. S1)

Supplementary Figure 2 (Fig. S2)

Supplementary Figure 3 (Fig. S3)

Supplementary Figure 4 (Fig. S4)

Supplementary Figure 5 (Fig. S5)

Supplementary Table 1 (Table. S1)


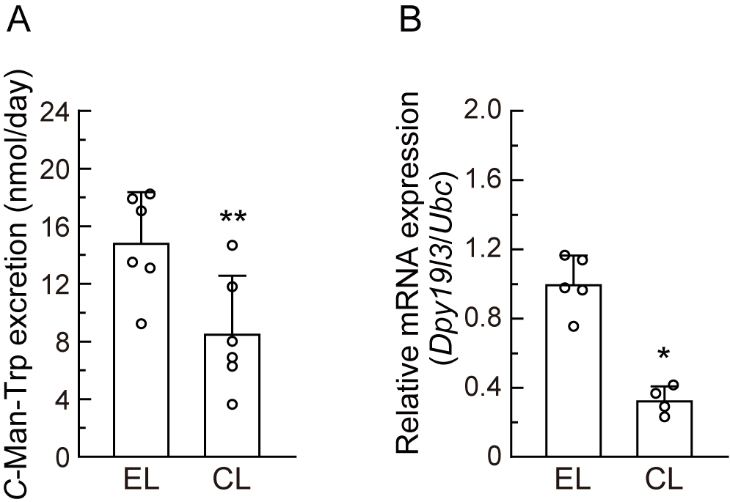


**Figure S1. Macrophage depletion decreased urinary *C*-Man-Trp excretion and *Dpy19l3* mRNA expression in PECs from mice with ovarian cancer.**

Female mice were intravenously injected with clodronate liposomes (CL) or empty liposomes (EL), and HM-1 cells were then transplanted into the peritoneal cavities of mice. A, Two days after transplantation, mice were kept in metabolic cages for 24 hours. Urine was collected and *C*-Man-Trp levels were measured. **P < 0.05 vs EL. B, Three days after transplantation, mice were sacrificed and the mRNA expression level of *C*-mannosyltransferase (*Dpy19l3*) in PECs was examined with RT-qPCR. *P < 0.01 vs EL.


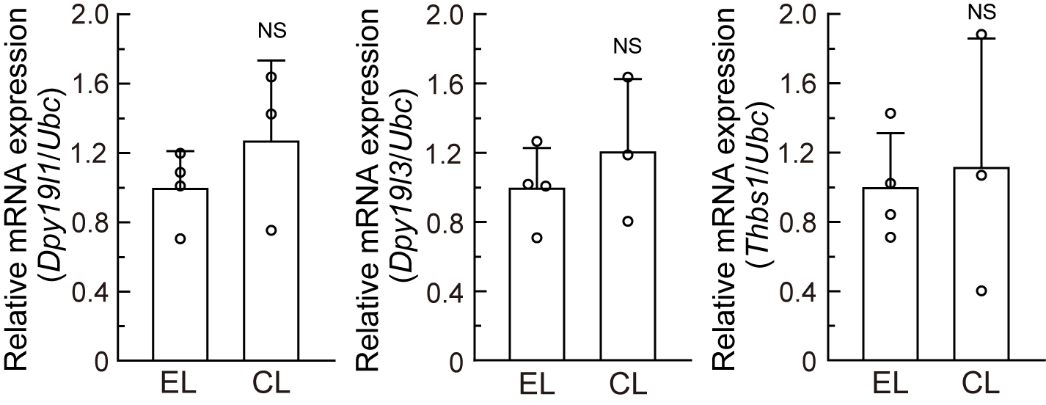


**Figure S2. Macrophage depletion did not affect *C*-Man-Trp metabolism-related gene expression in peritoneal tissue of mice with ovarian cancer.**

Female mice were intravenously injected with clodronate liposomes (CL) or empty liposomes (EL), and HM-1 cells were transplanted into the peritoneal cavities of mice. Five days after HM-1-cell transplantation, CL or EL was intravenously injected on day 5. On day 10 after transplantation, peritoneal tissues were collected and *C*-Man-Trp metabolism-related gene expression was analyzed by RT-qPCR. NS; not significant vs EL.

**

**

**Figure S3. Macrophage depletion did not affect *Dpy19l3* mRNA expression in peritoneal cavity cells of normal healthy female mice.**

Female mice were intravenously injected with clodronate liposomes (CL) or empty liposomes (EL). Five days later, peritoneal cavity cells were collected and *Dpy19l3* mRNA expression was analyzed by RT-qPCR. NS; not significant vs EL.

**

**

**Figure S4. Macrophage depletion did not affect *Dpy19l3* mRNA expression in liver tissues of normal healthy female mice.**

Female mice were intravenously injected with clodronate liposomes (CL) or empty liposomes (EL). Five days later, liver tissues were collected and *Dpy19l3* mRNA expression was analyzed by RT-qPCR. NS; not significant vs EL.

A B

**

**

**Figure S5. Macrophage depletion did not affect *C*-Man-Trp levels in the bone marrow or kidney of normal healthy mice.**

Female mice were intravenously injected with clodronate liposomes (CL) or empty liposomes (EL). Five days later, bone marrow (A) and kidney (B) tissues were collected and *C*-Man-Trp levels were measured. NS; not significant vs EL.

**Table S1 Primers used for RT-qPCR in the present study**

| Target |  | Primer Sequence (5’ → 3’) | Accession Number | Product Size (bp) |
| --- | --- | --- | --- | --- |
| Ccn1 | F | CTGAAGAGGCTTCCTGTCTTT | NM_010516 | 145 |
|  | R | TGGTAACTCGTGTGGAGATGC |  |  |
| Dpy19l1 | F | AATGATATGCGGGGTGTCGT | NM_001359948 | 113 |
|  | R | CCAAGGGCTGTGTACGCTAA |  |  |
| Dpy19l3 | F | CGTTCTCACCCTGCTGTACC | NM_178704 | 138 |
|  | R | ACAGCCTTCCTTGGTGTGTT |  |  |
| Spon1 | F | GAGAGATACGTGAAGCAGTTCC | NM_145584 | 179 |
|  | R | ATACGGTGCCTCTTCTTCATAC |  |  |
| Thbs1 | F | CAAGGGCTCAGGGATACTCA | NM_011580 | 180 |
|  | R | TGAGACGCCATCTGTATGCA |  |  |
| Ubc | F | CGTCGAGCCCAGTGTTACCACCAAGAAGG | NM_019639 | 112 |
|  | R | CCCCCATCACACCCAAGAACAAGCACAAG |  |  |
